# 

**Published:** 1887-01-29

**Authors:** 


### THE HOSPITAL

An Institution, Family, and Parochial Journal of
Hospitals, Asylums, & all Agencies for the Care of the Sick, Criticism & News.

### CONTENTS.

**Hospital Housekeeping** - - - - - 293
**Words of Consolation.**—XVIII. Sympathy with Nurses - - - - - - - - - 294
**Poetry.**—The One Law of Life - - - - - 294
**Hospitals, The Sick Poor, and All England.** By Henry C. Burdett - - - - - - 295
**The Story of the Hospitals.**—The British Home for Incurables (*continued*) - - - - - 296
**A Noble Profession: Nursing the Sick.**—The All Saints Sisters' Home for Trained Nurses. By Miss Simpson - - - - - - - 297
**Flowers, Ferns, and Ward Decorations** - 298
**America and its Charities: A Suggestive Experience** - - - - - - 299
**Notes and News.**—Miscellaneous Items.—Presentation to the Founders of the Stanley Hospital.—Hospital Reports.—Vacancies.—The Great Northern Central Hospital.—Kensington District Nursing Association.—Proposed Hospital for Mine Women, etc., etc. - 300
**Annotations.**—The Battle of St. Albans.—Hospital Sunday in Birmingham.—*The Spectator* and Vivisection - 303
**Reminiscences of the Insane.** By a Mental Physician - - - - - - - 304
**Poor-law Infirmaries and General Hospitals.**—Discussion on Colonel Montefiore's Paper - 305
**Till the Doctor Comes.**—IX. Sick Appliances for the Household - - - - - - 306
**Clothing and Health** - - - - - 307
**Disorders of Digestion.**—The Mouth - - 308
**The Prize Competitor** - - - - - 309
**The Children's Corner.**—Puzzles, Answers, etc. - 309
**In- and Out-Patients' Hospital Diary** - - 310

